# Rewiring of primary metabolism for ammonium recycling under short-term low CO_2_ treatment – its implication for C_4_ evolution

**DOI:** 10.3389/fpls.2024.1322261

**Published:** 2024-08-01

**Authors:** Fenfen Miao, Ying Wang, Noor UI Haq, Ming-Ju Amy Lyu, Xin-Guang Zhu

**Affiliations:** ^1^ University of Chinese Academy of Sciences (UCAS), Beijing, China; ^2^ CAS Center for Excellence in Molecular Plant Sciences, Institute of Plant Physiology and Ecology (SIPPE), Chinese Academy of Sciences (CAS), Shanghai, China; ^3^ Department of Computer Science and Bioinformatics, Khushal Khan Khattak University, Karak, Khyber-Pakhtunkhwa, Pakistan

**Keywords:** low CO_2_, photorespiration, ammonium refixation, regulatory preconditioning, C_4_ photosynthesis

## Abstract

The dramatic decrease in atmospheric CO_2_ concentration during Oligocene was proposed as directly linked to C_4_ evolution. However, it remains unclear how the decreased CO_2_ concentration directly facilitate C_4_ evolution, besides its role as a selection pressure. We conducted a systematic transcriptomics and metabolomics analysis under short-term low CO_2_ condition and found that *Arabidopsis* grown under this condition showed 1) increased expression of most genes encoding C_4_-related enzymes and transporters; 2) increased expression of genes involved in photorespiration and pathways related to carbon skeleton generation for ammonium refixation; 3) increased expression of genes directly involved in ammonium refixation. Furthermore, we found that *in vitro* treatment of leaves with NH_4_^+^ induced a similar pattern of changes in C_4_ related genes and genes involved in ammonium refixation. These data support the view that *Arabidopsis* grown under short-term low CO_2_ conditions rewired its metabolism to supply carbon skeleton for ammonium recycling, during which process the expression of C_4_ genes were up-regulated as a result of a hitchhiking process. This study provides new insights into the adaptation of the C_3_ model plant *Arabidopsis* under low CO_2_ conditions and suggests that low CO_2_ can facilitate the evolution of C_4_ photosynthesis beyond the commonly assumed role of being a selection pressure.

## Introduction

Although C_4_ plants only have 8000 species, but it contributes to 23% of the earth’s terrestrial primary productivity (Still et al., 2003; Sage et al., 2007). With over 60 distinct evolutionary origins (Sage et al., 2011; Sage et al, 2018), C_4_ photosynthesis represents an excellent example of convergent evolution, which attracts attentions among biologists to study the evolutionary history of C_4_ photosynthesis (Sage, 2001; Sage et al., 2011). According to the present models of C_4_ evolution (Sage, 2011; Sage, 2012), relocation of photorespiratory CO_2_ release to the bundle sheath cell (BSC) from the mesophyll cell (MC) is an important early step during the evolution of the C_4_ photosynthesis (Vogan et al., 2007; Sage, 2012). This step results in C_2_ photosynthesis, which is characterized by a photorespiratory CO_2_ pump can elevate the concentration of CO_2_ in BSC by ~ threefold (Keerberg et al., 2014). Immunogold experiments show that glycine decarboxylase (GDC) is expressed exclusively in the BSC of some intermediates, such as *Moricandia arvensis* (Rawsthorne et al., 1988; Schluter et al., 2017) and *Heliotropium* (Vogan et al., 2007). Due to the enrichment of CO_2_, Ribulose-1,5-bisphosphate carboxylase/oxygenase (Rubisco) works more efficiently in the BSC, leading to an overall increase in biomass production under condition which supports higher rates of photorespiration (Heckmann et al., 2013; Mallmann et al., 2014), such as low CO_2_, drought and high temperature (Wingler et al., 1999; Carmo-Silva et al., 2008; Li Y. et al., 2014; Sage et al., 2018).

Accompanied with the release of CO_2_ by GDC during photorespiration, ammonium (NH_4_^+^) is also released (Bauwe et al., 2010), which needs to be refixed since it is toxic (Krogmann et al., 1959). Photorespiratory ammonium can be refixed and integrated into amino acids via the chloroplastic isoform Glutamine synthetase 2 (GS2) and ferredoxin-dependent GOGAT (Fd-GOGAT) (Lam et al., 1996). This process forms the photorespiratory ammonium cycle (Keys, 2006). Photorespiratory CO_2_ pump results in ammonium imbalance between MC and BSC, which needs to be solved through metabolic shuttles (Nagatani et al., 1971; Lancien et al., 2000; Liu and von Wiren, 2017). Mallman (Mallmann et al., 2014) showed that there are multiple solutions to the ammonium imbalance, with one option, an alanine-pyruvate shuttle, providing a bridge toward C_4_ photosynthesis. Therefore, ammonium refixation induced by high rate of photorespiration was proposed a bridge toward C_4_ evolution. However, it raises a question regarding how these metabolic shuttles which recycle ammonium emerge during C_4_ evolution. Considering that these metabolic shuttles for ammonium recycling require a rather large scale rewiring of the plant’s primary metabolism, quickly evolving such a mechanism when ammonium imbalance in C_2_ plants appeared would be rather difficult. Importantly, C_4_ photosynthesis mostly emerged around 30 million years ago and originated independently more than 60 times (Sage et al., 2011), there seems little likelihood for such complex metabolic pathways and its associated regulatory mechanisms to evolve multiple times in such a short time.

All genes involved in C_4_ photosynthesis are present in C_3_ ancestral plants and play important house-keeping roles (Aubry et al., 2011); similarly, all the genes involved in the ammonium recycling already exist in gymnosperm species (Valderrama-Martín et al., 2022). It however remains to be tested whether there are regulatory mechanisms coordinating the expression of these genes under conditions favoring photorespiration in typical C_3_ plants.

The 30-million-year time span subsequent to the Oligocene CO_2_ decrease during which numerous C_4_ plants originated suggests that historically low CO_2_ levels most likely facilitated C_4_ evolution (Ehleringer et al., 1991; Ehleringer et al, 1997; Christin et al., 2008). Low CO_2_ condition enhances photorespiration (Li Y. et al., 2014). However, there is little study so far on how this low CO_2_ directly contributes to the evolutionary formation of the C_4_ pathway genetically. In this study, we attempt to answer this question through combined transcriptomics and metabolomics analysis. Given that under short-term low CO_2_ treatment, changes in enzymatic sequences would not be expected, this study focuses on test whether low CO_2_ altered expressions of genes related to C_4_ photosynthesis and how. Our results suggest that low CO_2_ condition indeed induced metabolic rewiring to increase ammonia refixation in the C_3_ model plant *Arabidopsis* thaliana, though the signaling pathways through which low CO_2_ induced observed changed gene expression remains unknown. Since many reactions involved in the ammonia refixation overlap with those for C_4_ photosynthesis, the mechanisms responsible for the up-regulation of genes related to ammonia refixation inevitably facilitated the up-regulation of genes related to C_4_ photosynthesis. Therefore, the up-regulation of C_4_ photosynthesis represents an excellent example of hitchhiking process for the emergence of an important biological process.

## Materials and methods

### Plant material and growth conditions

*Arabidopsis* used in this study is the Columbia (Col-0) ecotype. Seeds were surface-sterilized by incubation with 10% (v/v) bleach diluted with ethanol, washed three times with sterile distilled water, and then sown in plastic Petri dishes (diameter 90 mm, depth 20 mm) containing 1/2 MS medium, solidified with 0.7% (w/v) agar and supplemented with 1.5% (w/v) sucrose (pH 5.8). The seeds were placed in the dark at 4°C for three days and germinated for six days in incubators with a photosynthetic photon flux density (PPFD) of 100 μmol m^-2^s^-1^ (10 h light/14 h dark cycle) at 22°C. After this, seedlings were transferred to *Pindstrup* soil and grown in a Percival incubator (Nihonika, Japan) in which CO_2_ gas was accurately and stably controlled.

### Low CO_2_ treatment

The CO_2_ condition of 100 ppm (low CO_2_, LC) and 400 ppm (normal CO_2_, NC) were applied in two separate Percival incubators (Nihonika, Japan) and maintained throughout this study. *Arabidopsis* were grown for 14 days under a PPFD of 100 μmol m^-2^s^-1^ (10 h light/14 h dark cycle) at 22°C and relative humidity of 65% (Li Y. et al., 2014). After, half of the plants were transferred to low CO_2_ condition for six days. New fully expanded leaves in three-week-old plants were sampled at 11 am in the morning for transcriptomic and metabolomics analysis, the measurement of chlorophyll content and free ammonium levels under both low CO_2_ and normal CO_2_ levels.

### Ammonium treatment

To study the effect of ammonium ions on gene expression, seeds were surface-sterilized and sown in a control check (CK) plastic Petri dish (diameter 90 mm, depth 20 mm) in a growth chamber for 10 days at a PPFD of 100 μmol m^-2^s^-1^ (10 h light/14 h dark cycle) at 22°C. The composition of the CK was described in (Li et al., 2013), which is composed of 2 mM KH_2_PO_4_, 5 mM NaNO_3_, 2 mM MgSO_4_, 1 mM CaCl_2_, 0.1 mM Fe-EDTA, 50 μM H_3_BO_3_, 12 μM MnSO_4_, 1 μM ZnCl_2_, 1 μM CuSO_4_, 0.2 μM Na_2_MoO_4_, 0.5 gl^-1^ MES, 1% sucrose, and 0.8% agarose (pH 5.7, adjusted with 1 M NaOH). Half of the seedlings were transferred to the new CK petri dish and the remaining seedlings were transferred to new CK plates which were additionally supplied with 15 mM or 30 mM (NH_4_)_2_SO_4_. Nine seedlings were put on one Petri dish and considered as one biological replicate during sampling. After three days of treatments, leaves were harvested and immediately put into liquid nitrogen and stored at –80°C until RNA extraction. We also put seedlings on the CK plates which were additionally supplied with 30 mM or 60 mM NH_4_Cl, 15 mM or 30 mM K_2_SO_4_, 30 mM or 60 mM KNO_3_, and 120 mM mannitol to test the effect of other ions (Li et al., 2019).

### Measurement of free ammonium content in a leaf

Ammonium content was determined with the colorimetric method based on the phenol hypochlorite assay (Solórzano, 1969; Sarasketa et al., 2014). Briefly, leaves were collected, weighed, and snap-frozen in liquid nitrogen, and then ground with 5 mm zirconia beads. 1 mL H_2_O was added to the frozen powder. The mixtures were incubated at 80°C for 10 min and then centrifuged at 4000 g and 4°C for 20 min. 100 µL of supernatant was mixed with 200 μL of 0.33 M sodium phenolate, 100 µL of 0.02% sodium nitroprusside; then 200 µL of 2% sodium hypochlorite were added to the mixture. The mixture was incubated at room temperature for 30 min followed by reading absorbance at a wavelength of 630 nm in a spectrophotometer.

### Gene expression analysis

For gene expression profiling, we took illuminated new fully expanded leaf samples. Four biological replicates were harvested and frozen in liquid nitrogen immediately and stored at –80°C before RNA extraction. For RNA extraction, the PureLink RNA Mini Kit (Life Technologies Corporation, USA) was used to extract RNA following the manufacturer’s protocol, which includes the removal of gDNA from RNA. The quality of purified RNA was assessed using an Agilent 2100 Bioanalyzer (Agilent, USA) and the RNA samples with RNA Integrity Number (RIN) higher than 7 were used for RNA library construction. RNA libraries were prepared based on standard Illumina (Illumina, Inc., USA) protocols and sequenced using the Illumina X Ten platform in paired-end 150 bp mode. The quality of RNA-seq data (fastq files) was assessed by the FastQC software (https://www.bioinformatics.babraham.ac.uk/projects/fastqc/). RNA-seq analysis was performed by the STAR software (Dobin et al., 2013) with the *Arabidopsis* reference genome as well as a gene transfer format (GTF) file (downloaded from EnsemblPlants http://plants.ensembl.org/). After generation of the genome index, RNA-seq reads were aligned by STAR with the ‘–quantMode GeneCounts’ option to count reads per gene. Differentially expressed genes (DEGs) were determined by the R package ‘DESeq2’ (Love et al., 2014) which use the Benjamini-Hochberg (BH) adjustment for multiple testing problem with the read counts reported by STAR (Dobin et al., 2013). DEGs between treatment and control were analyzed. Only genes with the adjusted *P*-value <0.05 (Padj) were considered as DEGs in each treatment.

### Functional analysis of differentially expressed genes

Gene transcript abundances and metabolite abundances were visualized applying R package ‘heatmap’ (https://cran.r-project.org/web/packages/pheatmap/index.html). Permutation of Pearson correlations was conducted used the R package Permutation test. Volcano plot and Venn diagrams were plotted applying online tool Bioinformatics (https://www.bioinformatics.com.cn). To identify functional categories of differentially expressed genes, Gene Oncology (GO) and Kyoto Encyclopedia of Genes and Genomes (KEGG) pathway enrichment analyses were performed using the Database for Annotation, Visualization and Integrated Discovery (DAVID) (https://david.ncifcrf.gov/). Data were analyzed using the two-tailed Student’s *t-test*. The term significant is used here for differences or correlations confirmed at * P < 0.05 or better. Details are provided in the figure legends.

### Liquid chromatography/mass spectrometry and metabolomics analysis

To enable the sampling of leaves grown under low CO_2_, we modified the glass door of a Percival incubator to ensure the stability of CO_2_ condition during leaf sampling. Specifically, we cut two spherical openings in the middle of the glass door and installed two plastic gloves used for sampling.

The Liquid Chromatography/Mass Spectrometry (LC-MS/MS) experiments were performed following (Wang et al., 2014; Arrivault et al., 2019). Fully expanded leaves under light were sampled. For each sample, a leaf area of 1.7 cm^2^ was sampled and frozen in liquid nitrogen instantaneously. The same leaf position was sampled to measure the fresh weight to calculate specific leaf weight. All leaf samples were cut *in situ* and immediately transferred into a pre-frozen 2 mL EP tube, then stored in liquid nitrogen for metabolite extraction. After grinding, each sample was fully dissolved with 800 μL extraction buffer (methanol: chloroform = 7:3 (v/v), -20 °C pre-cooling) and incubated under -20°C for 3 hours. Then 560 μL distilled water (ddH_2_O) was added and mixed with each sample, and 800 μL supernatant was extracted after centrifugation (×2200g, 10min, 4°C). After that, 800 μL buffer (methanol: ddH_2_O = 1: 1(v/v), 4 °C pre-cooling) was mixed with the sample for another extraction. For each sample, 1.6 mL supernatant in total was obtained by filtering the extraction buffer with a 0.2 μM nylon filter. Among them, 1 mL was used for MS/MS analysis, and 20 μL was used for the QC sample. All extraction operations were performed on ice.

Luna NH_2_ column (3μm, 100mm*2mm, Phenomenex co. Ltd, USA) was used in the liquid chromatography. The Liquid Chromatography gradient was set with eluent A, which has 10 mM Ammonium acetate and 5% (v/v) acetonitrile solution, with the pH adjusted to 9.5 using ammonium water and eluent B (acetonitrile): 0-1 min, 15% A; 1-8 min, 15-70% A; 8-20 min, 70-95% A; 20-22 min, 95% A; 22-25 min, 15% A. During the mass spectrometry analysis, QTRAP 6500+ (AB Sciex, co. Ltd, USA) was used in the MRM model with all parameters used following (Wang et al., 2014; Arrivault et al., 2019). The condition of all metabolites in samples were calculated based on the “condition-peak area” curve of standard samples and converted to nmol·gFW-1 with specific leaf weight (Arrivault et al., 2019).

### Real-time RT–qPCR

The RT–qPCR analysis was conducted as described previously (Chen et al., 2020). The RNA was extracted with the same procedure as described in Gene expression analysis. For reverse transcription, 0.2 μg RNA was reverse transcribed with TranScript One-Step gDNA Removal and cDNA Synthesis SuperMix (TransGen Biotech, China), then SYBR Green Real-Time PCR Master Mix (Yeasen, China) was used for qPCR on the CFX 96 system (Bio-Rad) following the manufacturer’s protocol. The ACTIN 8 (AT1G49240) gene was used as a reference for mRNA normalization. The comparative cycle threshold (Ct) method was used to evaluate the relative gene expression levels. The primers used for the expression analysis are listed in **Supplementary Table S1**.

### Measurement of chlorophyll content

Leaf discs (0.8 cm^2^) were taken from fully expanded leaves, and chlorophyll was extracted with 80% acetone at 4°C for 24 h in darkness, then the supernatant was used for the absorbance measurement at 652 nm in a spectrophotometer (Arnon, 1949). The total chlorophyll content was calculated with the following formula:


$$\text{Total chlorophyll }(\text{μg}/{\text{cm}}^{2})=34.5\times \text{OD}652(\text{μg}/\text{mL})\times \text{V}(\text{mL})/\text{leaf area}({\text{cm}}^{2})$$

## Results

### The genes related in primary metabolism were induced under low CO_2_ condition

To study the effect of low CO_2_ condition on C_3_ plants, we examined the physiological, transcriptomic, and metabolomic changes of *Arabidopsis* under two CO_2_ conditions: low CO_2_ at a concentration of 100 ppm (LC) and normal CO_2_ at a concentration of 400 ppm (NC). Plants grown under low CO_2_ conditions had lower biomass and decreased chlorophyll than those grown under normal CO_2_ (**Supplementary Figures S1A, B**).

The differentially expressed genes (DEGs) were identified between low CO_2_ and normal CO_2_ treatments (**Supplementary Dataset S1**). 2976 genes were upregulated and 2892 genes were downregulated (**Supplementary Figure S2A**). Gene Ontology (GO) enrichment analysis shows that hexose catabolic process, sugar-phosphatase activity, carbohydrate phosphatase activity, amyloplast, carotene biosynthetic and abscisic acid metabolic process were significantly enriched in upregulated genes (**Supplementary Figure S2B**; **Supplementary Dataset S2**), whereas sulfur compound and jasmonic acid-mediated signaling pathway were enriched in the downregulated genes (**Supplementary Dataset S2**).

In addition, the significantly upregulated genes were mainly participated in metabolic process, including biosynthesis of secondary metabolites, glycolysis/gluconeogenesis, starch and sucrose and carbon metabolism by KEGG analysis (**Supplementary Figure S2C**; **Supplementary Dataset S2**). These results show that low CO_2_ induced changes to gene expression involved in primary metabolism.

### Most C_4_ related genes are upregulated under low CO_2_ condition in *Arabidopsis*

**Figure 1:**
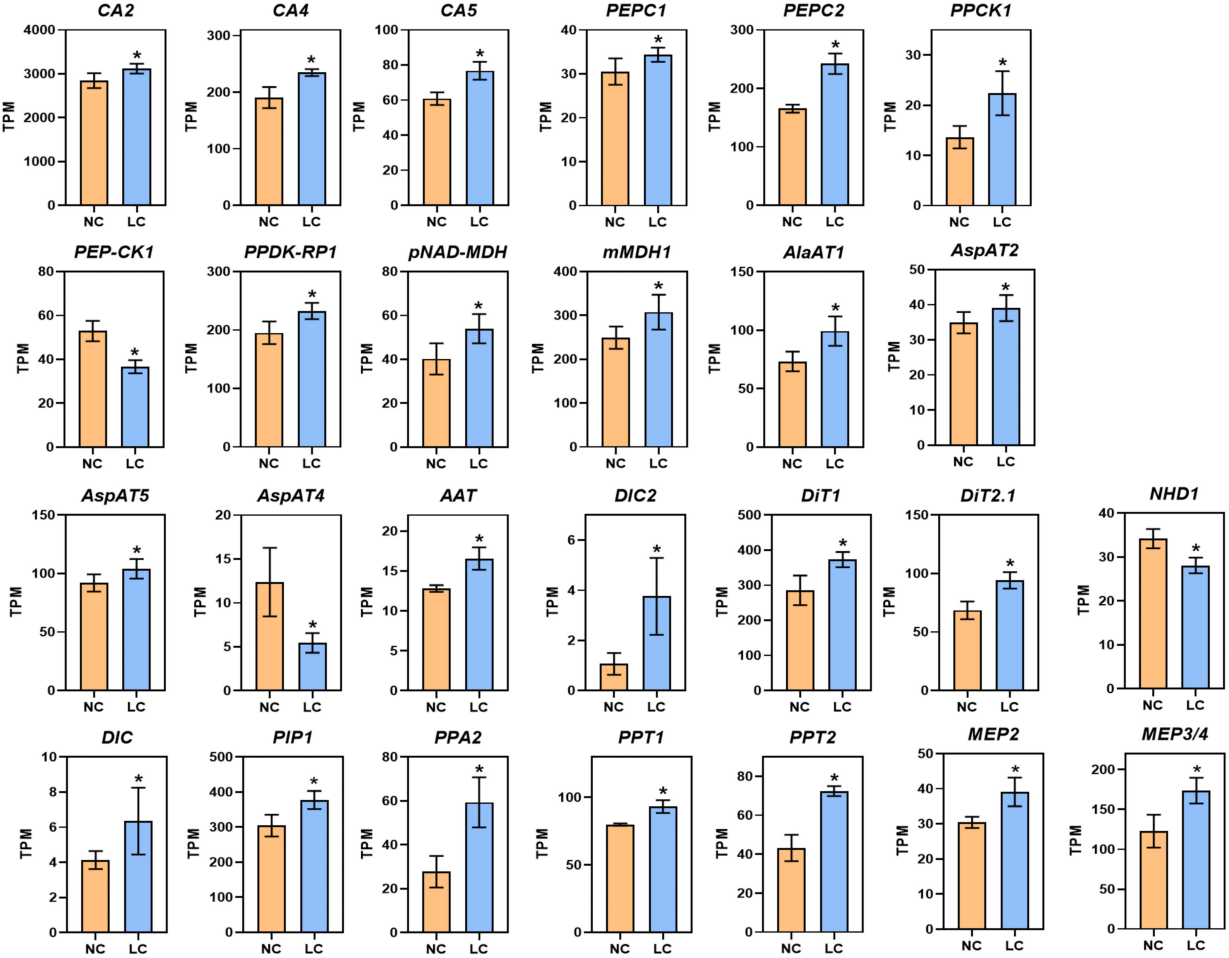
Low CO_2_ induction of C_4_ related genes in *Arabidopsis.* RNA-seq data are plotted as transcript per million (TPM). Gene expression is shown from samples collected from NC (normal CO_2_, 400 ppm) and LC (low CO_2_, 100 ppm). *CA2, Carbonic Anhydrase 2; CA4, Carbonic Anhydrase 4; CA5, Carbonic Anhydrase 5; PEPC1, Phosphoenolpyruvate Carboxylase 1; PEPC2, Phosphoenolpyruvate Carboxylase 2; PPCK1, PEPC Kinase1; PEP-CK1, Phosphoenolpyruvate Carboxykinase 1; RPDK-RP1, PPDK Regulatory Protein 1; pNAD-MDH, chloroplast NAD-Dependent Malate Dehydrogenase; mMDH1, mitochondrial Malate Dehydrogenase; AlaAT1, Alanine Aminotransferase1; AspAT4, Aspartate Aminotransferase 4; AspAT5, Aspartate Aminotransferase 5; AspAT2, Aspartate Aminotransferase 2; AAT, Aspartate Aminotransferase; PIP1, Plasma Membrane Protein; DIC2, Dicarboxylate Carriers 2; DiT2.1, Dicarboxylate Transport 2.1; DiT1, Dicarboxylate Transporter 1; DIC, Dicarboxylate Carriers; PPA2, Inorganic Pyrophosphatase 2; PPT1, Phosphoenolpyruvate Translocator 1; PPT2, Phosphoenolpyruvate Translocator 2; NHD1, Na+/H+ antiporter; MEP2, Mesophyll Envelope Protein 2; MEP3/4, Mesophyll Envelope Protein 3/4.* Data are shown as mean ± s.d (replications *n*=4). *, *adjusted P-value <*0.05 which was determined by DESeq2.

Notably, under low CO_2_, 23 of 26 genes associated with C_4_ metabolism were significantly increased (**Figure 1**; **Supplementary Dataset S1**), including cytoplasmic carbonic anhydrase (CA2, AT5G14740; CA4, AT1G70410) and chloroplast-localized carbonic anhydrase (CA5, AT4G33580). The gene expression of phosphoenolpyruvate carboxylase (PEPC, AT2G42600, AT1G53310), PEPC kinase (PPCK1, AT1G08650), chloroplast/mitochondrial NAD-dependent malate dehydrogenase (pNAD-MDH, AT3G47520; mMDH1, AT1G53240), pyruvate orthophosphate dikinase regulatory protein (PPDK-RP, AT4G21210), alanine aminotransferase (AlaAT1, AT1G17290), and aspartate aminotransferase (AspAT, AT4G31990, AT5G19550, AT2G22250) were also upregulated (**Figure 1**; **Supplementary Dataset S1**). In addition, the genes encoding C_4_ related transporters were upregulated significantly, which includes phosphoenolpyruvate/phosphate translocator (PPT1, AT3G01550; PPT2, AT5G33320), dicarboxylate carriers (DIC, AT4G24570; DIC2, AT2G22500), inorganic pyrophosphatase 2 (PPA2, AT2G18230), plasma membrane protein (PIP1, AT3G61430), and mesophyll envelope protein (MEP2, AT5G23890; MEP3/4, AT5G12470), dicarboxylate transporters (DIT1, AT5G12860; DIT2.1, AT5G64290). Many of these genes, such as PEPC, PPDK and NADP-ME, show increased gene expression under different stress conditions, such as high temperature, drought, high light, and saline conditions (Doubnerova and Ryslava, 2011). Overall, these results show low CO_2_ condition can cause up-regulation of genes involved in C_4_ photosynthesis.

We validated 12 genes that are involved in primary metabolism through RT-qPCR. The results of the RT-qPCR were consistent with the RNA-seq analysis as shown in **Supplementary Figure S3A**, with a Pearson correlation coefficient of 0.75 (**Supplementary Figure S3B**).

### Photorespiratory and ammonium refixation pathway are enhanced under low CO_2_ condition

**Figure 2:**
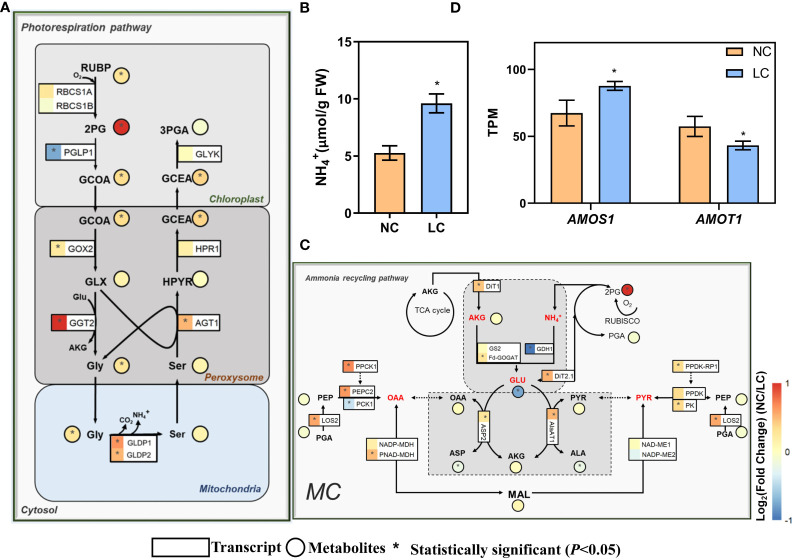
Changes of the gene expression and metabolites concentration on photorespiratory and ammonium recycling pathways under low CO_2_. **(A)** Photorespiratory pathway. **(B)** NH_4_^+^ content in the leaf (replications *n*=4). **(C)** Ammonium recycling pathway. **(D)** The changes of expression of ammonium-sensitive genes. AMOS1 (AT5G35220, *Ammonium-overly-sensitive 1*), AMOT1 (AT3G20770, *Ammonium tolerance 1*). The relative changes of gene expression and metabolites are shown in log2Fold change in heatmap, which use the same heatmap scale. For gene expression **(A, C, D)** differentially expressed genes (DEGs) were determined by the R package ‘DESeq2’ with the *adjusted P-value* <0.05 (replications *n*=4). For metabolites **(A, C)** and free NH_4_^+^ content in the leaf **(B)** which were determined by two-sided Student’s *t*-test (replications *n*=5) and * indicates *P*<0.05. Data are shown as mean ± s.d **(B, D)**. The symbols of genes in these metabolic pathways are from the *Arabidopsis* Information Resource (TAIR) Database (http://www.arabidopsis.org/), and also can get from **Supplementary Dataset S1**.

Photorespiration was enhanced under low CO_2_ condition (Li Y. et al., 2014). Photorespiratory ammonium refixation are highly integrated with photorespiratory metabolism (Keys et al., 1978). Therefore, we investigated the expression of genes related to photorespiration and ammonium refixation pathways. The expression of a number of photorespiratory genes, including glycolate oxidase (GOX2, AT3G14415), glutamate: glyoxylate aminotransferase (GGT2, AT1G70580), alanine: glyoxylate aminotransferase (AGT1, AT2G13360), glycine decarboxylase P-protein (GLDP1, AT4G33010; GLDP2, AT2G26080), and mitochondrial transporter A BOUT DE SOUFFLE (Bou, AT5G46800) (Eisenhut et al., 2013), were upregulated (**Figure 2A**). The gene coding for 2-phosphoglycolate phosphatase (PGLP1, AT5G36700) showed decreased expression (**Figure 2A**). Concurrent metabolomics analysis shows that the shift to low CO_2_ condition led to higher intracellular pools of 2-phosphoglycolate (2-PG), glycolate, glycine, and glycerate, which are intermediates in the photorespiratory pathway (**Figure 2A**). The increased metabolite levels corroborate with the upregulation of photorespiratory gene expression. In addition, the NH_4_^+^ levels in leaves were increased by one-fold compared to normal CO_2_ treatment (**Figure 2B**).

For ammonium refixation pathway, the gene coding Fd-GOGAT (AT5G04140) also showed significantly higher expression level under low CO_2_ condition (**Figure 2C**). DIT1 and DIT2.1 work in concert in photorespiratory carbon/nitrogen metabolism; the lack of either DiT2.1 or DiT1 led to growth impairment under photorespiratory condition (Kinoshita et al., 2011). In our data, both DiT2.1 and DitT1 showed greater expression levels under low CO_2_ (**Figure 2C**). Moreover, the gene expression of AspAT and AlaAT, which are involved in the transamination, showed significantly increase (**Figure 2C**). The concentration of 2-oxoglutarate (2-OG) showed no significant change (**Figure 2C**), whereas the concentration of glutamate was reduced by half (**Figure 2C**). The concentration of oxaloacetate (OAA) and pyruvate were not changed under low CO_2_ condition, while those of aspartate and alanine were slightly decreased (**Figure 2C**).

Under low CO_2_ condition, the expression of the gene *AMOS1* (*Ammonium-overly sensitive 1*) (Li et al., 2012), encoding a plastid metalloprotease that confers sensitivity to ammonium, significantly increased as depicted in **Figure 2D**. Conversely, the expression of AMOT1 *(Ammonium tolerance 1*) (Li et al., 2019), which is crucial for mitigating the effects of ammonium toxicity and confers greater tolerance to NH_4_^+^ in mutants than the wild type, was markedly reduced (**Figure 2D**). All these data suggest that *Arabidopsis* under low CO_2_ was subjected to ammonium stress under low CO_2_ condition.

### Low CO_2_ condition reprogramming primary carbon metabolism

**Figure 3:**
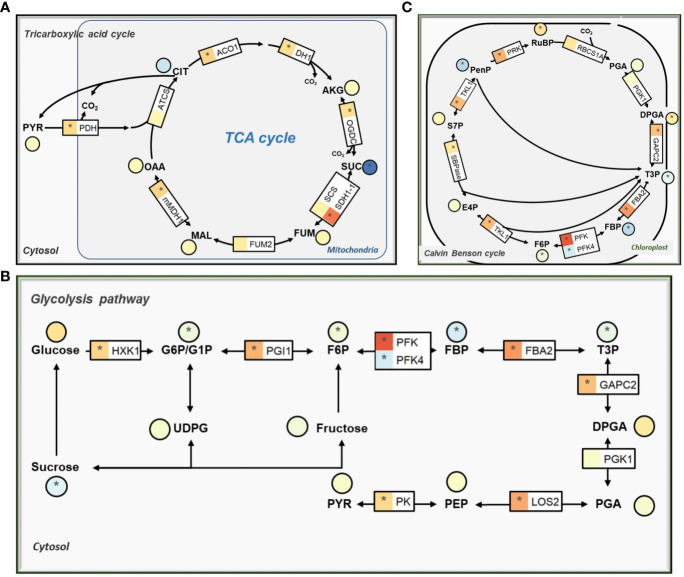
The effect of low CO_2_ on gene expression and metabolites condition in carbon metabolism pathways. **(A)** Tricarboxylic acid cycle (TCA cycle). **(B)** Glycolysis pathway. **(C)** Calvin-Benson-Bassham (CBB) cycle. The details are same as **Figure 2**.

The KEGG enrichment analysis of DEGs showed that low CO_2_ affects genes related to primary carbon metabolism (**Supplementary Dataset S2**). Among the affected genes, a vast majority of those involved in the tricarboxylic acid cycle (TCA) were markedly upregulated, specifically aconitase (ACO1, AT4G35830), isocitrate dehydrogenase 1 (IDH1, AT4G35260), 2-oxoglutarate dehydrogenase (OGDC, AT3G55410), succinate dehydrogenase (SDH, AT5G66760), and mMDH1 (**Figure 3A**; **Supplementary Dataset S1**). However, we observed a significant reduction of the succinate and fumarate in comparison to normal CO_2_ condition (**Figure 3A**).

The expression levels of genes associated with the glycolysis pathway showed similar trends of changes as those observed among the TCA cycle genes. Notably, most of these genes demonstrated significant upregulation while metabolites like sucrose, glucose-6 phosphate (G6P), fructose-6-phosphate (F6P), fructose-1:6-Bisphosphate (FBP), and triose phosphate (T3P) were significantly reduced under low CO_2_ treatment (**Figure 3B**). We observed increased expression levels of enolase (LOS2, AT2G36530), pyruvate kinase family protein (AT5G08570; AT2G36580), and pyruvate dehydrogenase (PDH-E1 BETA, AT1G30120; PDH-E1_ALPHA, AT1G01090). Among the genes related to the Calvin-Benson-Bassham (CBB) cycle, most of the genes showed upregulation in terms of expression, and the majority of the metabolites were affected (**Figure 3C**). While pentose phosphate (PenP), FBP, and T3P metabolites decreased significantly, other metabolites mostly showed an increase in their levels under the low CO_2_ condition (**Figure 3C**).

The 2-PG showed the strongest increase compared to other photorespiratory metabolites (**Figure 2**), which is in well agreement with the transcriptional repression of PGLP1 (**Figure 2**). Earlier work found that 2-PG inhibits CBB cycle enzymes TPI and SBPase in *Arabidopsis* after treatment with low CO_2_ (Flugel et al., 2017). Such an inhibition may decrease further increase of 2-PG and hence avoid potential greater damage to the cell under low CO_2_.

These results show that under low CO_2_ condition *Arabidopsis* changed expression of primary carbon metabolism and adjusted their metabolite to boost its adaptability to low CO_2_ condition.

### NH_4_^+^ treatment increased the expression of genes involved in ammonium refixation

**Figure 4:**
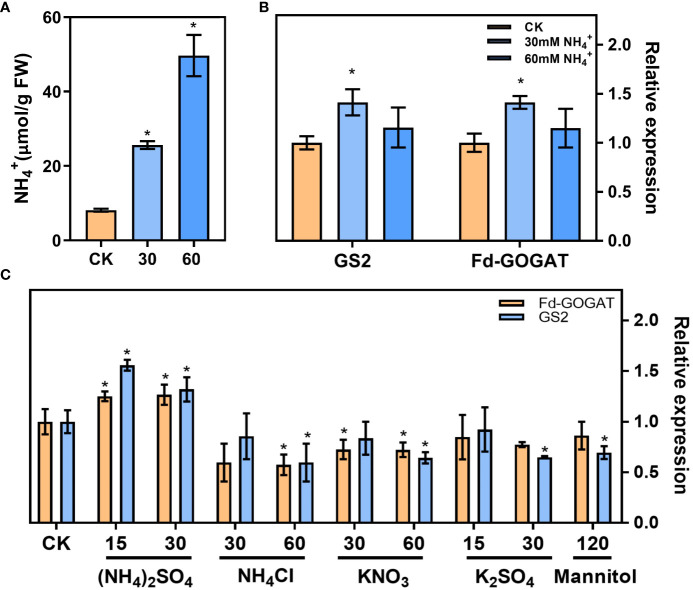
*In vitro* NH_4_^+^ treatment increased the expression of genes involved in ammonium refixation. **(A)** NH_4_^+^ content in the seedlings (Control check, CK; 30 mM NH_4_^+^, 30; 60 mM NH_4_^+^, 60) (replications *n*=4). **(B)** The gene expression of the Glutamine synthetase 2 (GS2) and ferredoxin-dependent GOGAT (Fd-GOGAT) were analyzed using RT–qPCR (replications *n*=4). **(C)** The effects of different ion on the gene expression of GS2 and Fd-GOGAT. Data are shown as mean ± s.d, * *p*<0.05, which were determined by two-sided Student’s *t-test*.

In our results, the leaf NH_4_^+^ content increased (**Figure 2B**) and the ammonium refixation was induced (**Figure 2C**) following low CO_2_ treatment. Plants treated with 30 mM NH_4_^+^ increased the leaf NH_4_^+^ content by at least one-fold, and the NH_4_^+^ content increased by at least three-fold with the 60mM NH_4_^+^ treatment (**Figure 4A**). RT-qPCR analysis revealed that NH_4_^+^ treatment upregulated the expression of genes involved in ammonium refixation, specifically GS2 and Fd-GOGAT as shown in **Figure 4B**. Interestingly, this increase was not observed when plants were treated with NH_4_CI, K_2_SO_4_, KNO_3_, or Mannitol (Li et al., 2019) (**Figure 4C**).

### Comparison of transcriptomics data between NH_4_^+^ treatment and low CO_2_ treatment

After NH_4_^+^ treatments, a total of 3920 genes were identified as differentially expressed with 1910 genes upregulated and 2010 genes downregulated (**Supplementary Figure S4A**). The GO terms for upregulated genes were enriched in stress, nitrogen component metabolism, carbohydrate biosynthetic process and oxoacid metabolism (**Supplementary Figure S4B**), which is in line with earlier finding that excessive NH_4_^+^ induced ammonium refixation related genes, such as glutamate dehydrogenase (Patterson et al., 2010; Daniela et al, 2016), and GS/GOGAT pathway (Liu and von Wiren, 2017). The upregulated genes were also significantly enriched in carbon metabolism and 2-oxocarboxylic acid metabolism (**Supplementary Dataset S2**), with latter term including pyruvate, oxaloacetate and 2-OG which can enter the TCA cycle to generate carbon skeleton for ammonium refixation.

**Figure 5:**
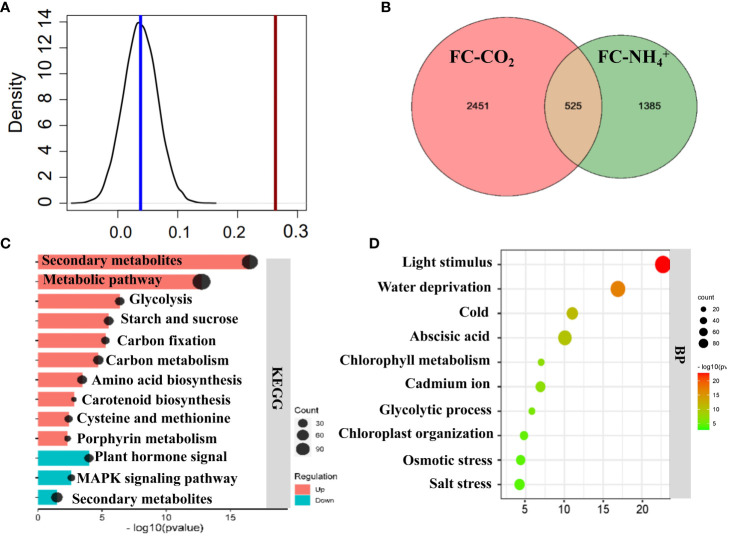
Comparison of transcriptomic profiling between NH_4_^+^ and low CO_2_ treatments. **(A)** Pearson correlation analysis of transcriptomics data between NH_4_^+^ and low CO_2_ treatment. The black line represents the distribution of Pearson correlations of randomly selected genes (1632) for 1000 times, and the red line represents the Pearson correlation of differentially expressed 1632 genes. A cutoff value for *adjusted P-value* < 0.05 were used to identify differentially expressed genes. **(B)** Venn diagrams. The diagram shows the number of genes which are jointly significantly upregulated (*adjusted P-value* < 0.05) under low CO_2_ treatment (FC-CO_2_) and NH_4_^+^ treatment (FC-NH_4_^+^). Detailed gene information can be found in **Supplementary Dataset S3**. **(C)** KEGG enrichment analysis of significantly upregulated genes. **(D)** Gene ontology (GO) Biological process (BP) enrichment analysis of significantly upregulated genes. The size of the circles represents the enriched gene number, with a larger circle indicating a higher number of genes in the corresponding metabolic pathway category. The horizontal axis represents the P-value. Top 10 significantly enriched results are shown here **(C, D)**. Remaining enriched results can be found in **Supplementary Dataset S3**. Fisher’s exact text *P<0.05*.

We compared the transcriptomic changes induced by both NH_4_^+^ and low CO_2_ treatments. We found that DEGs (*P<0.05*) under both treatments showed a higher Pearson correlation coefficient than those with no significant changes to either treatment (**Figure 5A**) (*P<0.001*), which suggest that the treatment of NH_4_^+^ and low CO_2_ caused similar changes. The Venn diagram shows that 525 genes were upregulated under both treatments (**Figure 5B**; **Supplementary Dataset S3**), while 464 genes were downregulated under both treatments (**Supplementary Figure S4C**). The jointly upregulated genes are mostly enriched in glycolysis, starch and sucrose metabolism, carbon metabolism, accounting for 36% of those differentially expressed upregulated genes (**Figure 5C**). The jointly upregulated genes were also found to be enriched in biological processes related to light stimulation, water deprivation, cold, abscisic acid, and the glycolytic process (**Figure 5D**). These results suggest that multiple genes involved in primary metabolism were simultaneously affected by both NH_4_^+^ and low CO_2_ treatments.

### Influence of NH_4_^+^ treatment on the gene expression in primary metabolism

**Figure 6:**
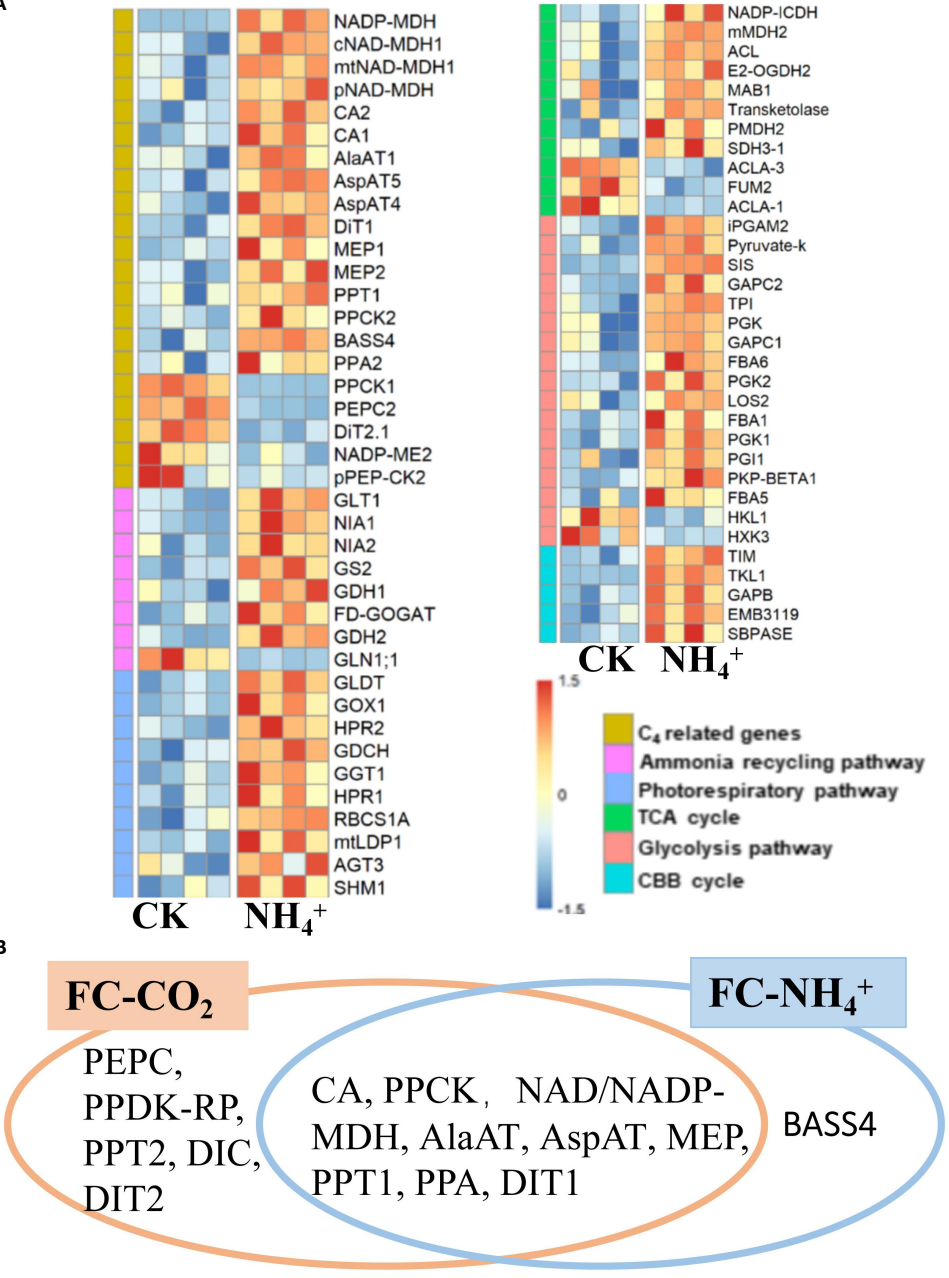
**(A)** Heatmap of differentially expressed genes of different metabolic pathways under NH_4_^+^ treatment (replications *n*=4). Genes from six pathways were shown as labeled in different colors indicated in the “Pathways” legend. **(B)** Comparative analysis of C_4_ related enzymes under low CO_2_ treatment (FC-CO_2_) and NH_4_^+^ treatment (FC-NH_4_^+^). The fisher’s exact text *P<0.05*.

Transcriptomic data from plants treated with NH_4_^+^ showed an increase in expression levels of the GS/GOGAT pathway and NADH-dependent glutamate dehydrogenase (GDH), as well as upregulation of nitrate reductase genes (NIA1, AT1G77760; NIA2, AT1G37130) (Masclaux-Daubresse et al., 2010) (**Figure 6A**), suggesting that NH_4_^+^ treatment enhances ammonium refixation metabolism. Additionally, RNA-seq data showed that almost all genes related to the photorespiratory pathway were induced, along with the expressions of genes related to the TCA cycle, Glycolysis pathway, and the CBB cycle (**Figure 6A**). The transcriptomic results in primary metabolism induced by NH_4_^+^ treatment were similar to those induced by low CO_2_ treatment (**Supplementary Figure S5**).

Further analysis suggested that NH_4_^+^ treatment induced the expression of most C_4_ related genes, including CA (CA1, AT3G01500; cytoplasmic CA2, AT5G14740), NADP-dependent malate dehydrogenase (NADP-MDH, AT5G58330), cytosolic malate dehydrogenase (cMDH1, AT1G04410), NAD-MDH1 (AT1G53240, AT3G47520), and phosphoenolpyruvate carboxylase kinase (PPCK2, AT3G04530) (**Figure 6A**; **Supplementary Dataset S1**). Furthermore, some aminotransferases, such as AspAT4, AspAT5 (AT4G31990), and AlaAT1 were up-regulated by NH_4_^+^ treatment (**Figure 6A**; **Supplementary Dataset S1**). Several transporter genes, including DiT1, PPT1, Sodium Bile acid symporter (BASS4, AT3G56160), PPA2, NHD1, and MEP, were also upregulated by NH_4_^+^ treatment (**Figure 6A**; **Supplementary Datasets S1**). However, the expression levels of PEPC, PPCK1, and pPEP-CK2 were downregulated (**Figure 6A**). In addition, we also found that CA, NAD/NADP-MDH, PPCK, AlaAT, AspAT, MEP, PPT1, PPA, and DIT1 were significantly induced by both treatments (**Figure 6B**). Furthermore, low CO_2_ treatment induced the upregulation of most C_4_ related genes, almost including those induced by NH_4_^+^ treatment, except for BASS4 (**Figure 6B**) (Fisher’s exact text *P<0.05*). Therefore, NH_4_^+^ accumulation may be a mechanism that upregulates C_4_ genes under condition of low CO_2_ (**Figure 6B**).

## Discussion

### The gene expression involved in the primary metabolism is adjusted to increase the capacity of ammonium refixation under low CO_2_ condition

*Arabidopsis* grown under low CO_2_ condition showed a significant decrease in growth (Li Y. et al., 2014) and chlorophyll content compared to the control (**Supplementary Figures S1A, B**). The stunted growth of *Arabidopsis* under low CO_2_ condition can be attributed to the decreased photosynthetic CO_2_ fixation. The observed decrease in chlorophyll concentration may be related to nitrogen deficiency, as chlorophyll concentration depends on nitrogen content (Ercoli et al., 1993). The high photorespiration under low CO_2_ leads to an excessive amount of ammonium release in the leaves, which might result in decreased nitrogen content and chlorophyll concentration. Carbon starvation also reduces the capacity of plants to assimilate nitrogen, leading to significant reductions in leaf nitrogen and Rubisco capacity (Dippery et al., 1995; Sage and Coleman, 2001), disrupted C/N ratio (Tcherkez et al., 2017) and induction of autophagic responses (McLoughlin et al., 2020). In our study, carbon starvation resulted in decreased sucrose levels in leaves (**Figure 3C**). Interestingly, the expression of genes related to amino acid biosynthesis, carbohydrate biosynthesis, carotenoid biosynthesis *etc.* were increased (**Figure 5C**). This might be a compensatory effect, which has also been observed in studies of carbon-starved maize (McLoughlin et al., 2020).

The upregulation of PEPC and PEPE-K genes in response to low CO_2_ has also been observed in our earlier study (Li Y. et al., 2014). In this new study, we found that the expression of almost all core C_4_ cycle enzymes and transporters were upregulated under low CO_2_ treatment (**Figure 1**). Interestingly, a similar upregulation of C_4_ like pathway genes for carbon fixation was observed in *Nannochloropsis oceanica* under low CO_2_ condition (Wei et al., 2019). These data suggest a role of low CO_2_ condition in the strengthening of C_4_ gene expression during the Oligocene period when C_4_ emerged genes during evolution.

Low CO_2_ condition, like other stresses such as drought and high temperature (Carmo-Silva et al., 2008; Peterhansel and Maurino, 2011), increases photorespiration (**Figure 2A**) and promotes NH_4_^+^ production (**Figure 2B**) (Frantz et al., 1982; Alencar et al., 2019). Photorespiration is the primary source of NH_4_^+^ in higher plants (Leegood et al., 1995; Kumagai et al., 2011). *Arabidopsis*, being an NH_4_^+^ sensitive plant (Cruz et al., 2006), showed stunted plant growth and leaf chlorosis under low CO_2_ (**Supplementary Figures S1A, B**) (Li et al., 2012; Li Y. et al., 2014; Esteban et al., 2016). Taken together, these results suggest that *Arabidopsi*s experiences ammonium stress under low CO_2_ condition.

Previous studies have shown that excess ammonium is toxic (Hachiya et al., 2012; Li B. et al., 2014; Esteban et al., 2016) and needs to be refixed by upregulation of several key enzymes (Cruz et al., 2006; Liu and von Wiren, 2017; Hachiya et al., 2021). Indeed, the GS/GOGAT pathway which is involved in ammonium refixation was enhanced under low CO_2_ condition (**Figure 2C**). The TCA cycle under illumination helps provide the carbon skeleton, in particular 2-OG, for ammonium refixation (Nunes-Nesi et al., 2010; Sweetlove et al., 2010; Tcherkez et al., 2017). The gene expression of IDH1 and ACO1, which are used for the generation of 2-OG, were also upregulated in the TCA cycle (**Figure 3A**). Although the concentration of 2-OG remained unchanged (Arrivault et al., 2009), a significant reduction in the concentrations of fumarate and succinate were observed (**Figure 3A**), implying that there is an increased flux of 2-OG used for ammonium refixation, rather than decarboxylation in the TCA cycle. Consistent with this, studies have also shown that supplementing with succinate and 2-OG can increase ammonium recirculation and alleviate ammonium toxicity (Yuan et al., 2007; Hachiya et al., 2012).

**Figure 7:**
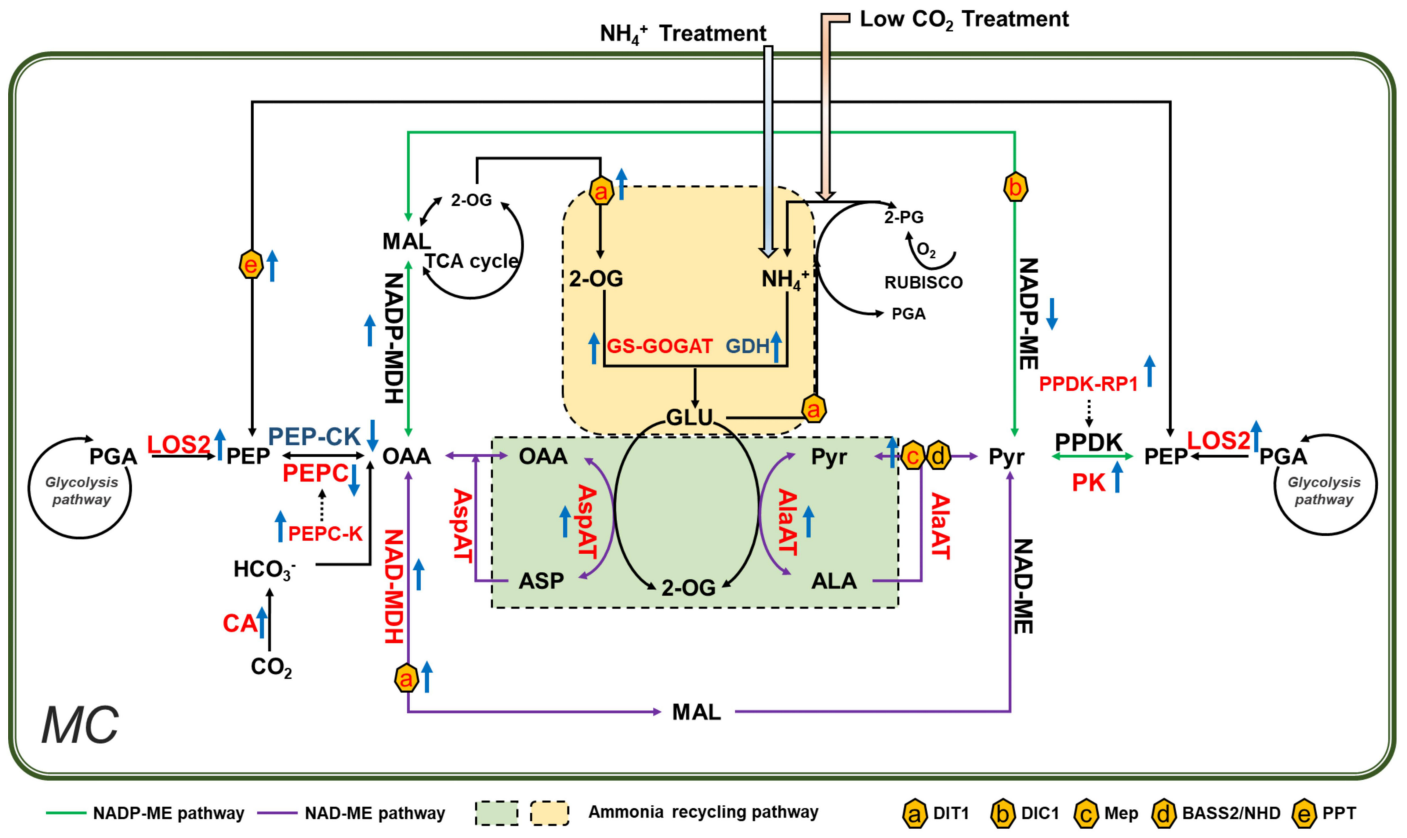
Ammonium accumulation and refixation is a primary mechanism inducing C_4_ related gene expression under low CO_2_ condition in *Arabidopsis*. Low CO_2_ treatment caused ammonium toxicity in *Arabidopsis*. Recirculation of ammonium requires reprogramming of primary metabolism reactions that involved in virtually all core C_4_ genes. *In vitro* NH_4_^+^ treatment induces a similar pattern of changes in C_4_ related genes and genes involved in primary metabolism. Therefore, we proposed that increased ammonium is a primary mechanism to induce the expression of C_4_ related gene under low CO_2_ in *Arabidopsis*. The Enzymes labeled in different colors were indicated as red/blue: upregulated/downregulated under low CO_2_. Rising/descending arrows beside the enzymes indicated upregulated/downregulated in NH_4_^+^ treatment. NADP-ME and NAD-ME pathways were represented in blue and purple lines respectively. Ammonium recycling pathways were shown in green and yellow rectangles; membrane transporters were indicated in hexagons.

Previous calculations suggest that the stored leaf citrate content available at the start of light period is insufficient to support the amount of 2-OG required for glutamate production (Stitt et al., 2002). The 2-OG can be generated by reactions catalyzed by aminotransferases. In this aspect, PEPC might play a crucial role during the synthesis of additional 2-OG (Turpin, 1994). Potatoes with overexpressed PEPC display increased 2-OG levels in the leaves (Rademacher et al, 2002); PEPC catalyzes the formation of OAA, which is used in transamination to produce 2-OG. In line with this, the genes encoding PEPC, PEPC-K, AspAT, and MDH are found upregulated under low CO_2_ condition (**Figure 7**). In addition, CAs, which are involved in optimizing cytosolic PEPC activity and ensure normal growth under low CO_2_ condition (DiMario et al., 2016; Weerasooriya et al., 2022), increased significantly under low CO_2_ condition (**Figure 1**). Similarly, the expression of PPDK-RP and AlaAT were upregulated, which can also be used to produce more pyruvate and carbon skeletons (**Figure 7**). Besides, the related transporters, such as Dit1, DIT2.1, showed increased expression as well, which might help meet the increased demand of metabolite transport (Gowik et al., 2011) (**Figure 7**). All these show that under low CO_2_ condition, the primary metabolism can be reprogrammed to generate the required carbon skeletons for ammonium refixation.

### NH_4_^+^ accumulation is a possible signal inducing change in the expression of C_4_ related genes under low CO_2_ in *Arabidopsis*

How could low CO_2_ induce the observed changes in transcriptomics? Here we discuss three possibilities. First, the observed change in transcriptomics might be directly caused by the ammonium accumulation. Indeed, the DEGs identified under low CO_2_ and NH_4_^+^ treatments show correlations (**Figure 5A**). Specially, under both low CO_2_ and NH_4_^+^ treatments, TCA cycle and related aminotransferases which provide 2-OG for ammonium refixation were significantly upregulated (**Figure 6A**; **Supplementary Figure S4**). Furthermore, the C_4_ related genes were induced by both low CO_2_ and NH_4_^+^ treatments (Test of independence using Fisher test, *P<0.05*) (**Figure 6B**). All these suggest that NH_4_^+^ accumulation can be a strong candidate signal inducing the expression of C_4_ related genes under low CO_2_ in *Arabidopsis*. However, the detailed mechanism of how ammonium induced these diverse responses is still unknown. Transcriptomic analysis reveals that among the genes activated in response to NH_4_^+^, 90% can be regulated by *AMOS1* (Li et al., 2012). Interestingly, the transcription level of *AMOS1* was increased under low CO_2_ (**Figure 2D**), suggesting *AMOS1* might be as a potential candidate to induce C_4_ related gene expression.

Secondly, low CO_2_ causes decreased photosynthesis, which resulted in decreased biomass production (**Supplementary Figure S1A**) and decreased concentrations of certain metabolites, such as sucrose (**Figure 3B**). In general, decreased concentration of carbohydrate enhances expression of genes for CO_2_ fixation for both C_3_ and C_4_ plants and also genes related to mobilization of photosynthesis reserve, and export process (Sheen, 1990; Cheng et al., 1992; Koch, 1996; Ruan, 2014). Given this, decreased sugar concentration might also be a potential mechanism underlining the observed reconfiguration of primary metabolism under low CO_2_ condition.

Finally, according to (You et al., 2020), ABA and PEPC play crucial roles in enabling *Arabidopsis* to adapt to low CO_2_ condition. Intriguingly, there was a notable increase in the expression of ABA related metabolic processes under low CO_2_ treatment (**Supplementary Dataset S2**) which implies that ABA signaling might have been involve in the observed changed in the transcriptomic under low CO_2_ condition as well.

### Implication of the existence of regulatory mechanisms inducing ammonium refixation for C_4_ evolution

One major question to answer in the field of C_4_ photosynthesis evolution is the detailed process through which rewiring of primary metabolism from C_3_ to C_2_ photosynthesis occurred. C_2_ photosynthesis, which is considered as an intermediate stage during C_4_ evolution (Bauwe et al., 2010; Heckmann et al., 2013), has been proposed to use ammonium recycling to resolve the issue of ammonium misbalance between BSC and MC (Mallmann et al., 2014). A number of putative pathways to recycle NH_4_^+^ were proposed by (Mallmann et al., 2014). These pathways usually use many, though not all, enzymes of the C_4_ cycle. In all these proposed pathways, ammonium is refixed by GS/GOGAT pathway with carbon skeleton provided by a partial C_4_ cycle. Several experimental results support this hypothesis. For example, in C_3_–C_4_ intermediate Flaveria species, the transcripts GS/GOGAT are upregulated, as also the case for a number of aminotransferases (Mallmann et al., 2014). The question now needs to answer is that, considering that C_4_ photosynthesis emerged from C_3_ photosynthesis in a relatively short geological time scale, i.e., only within about ~20 million years (Christin et al., 2008), how could C_3_ plants rapidly gain such complex regulatory mechanisms regulating ammonium recycling and providing carbon skeleton when the atmospheric CO_2_ drops at Oligocene?

Our results here show that the regulatory mechanism inducing ammonium recycling pathway pre-existed in C_3_ plants. Under low CO_2_ condition or other conditions where the photorespiratory flux and correspondingly the ammonium release flux are high, an innate regulatory mechanism to induce expression of genes involved in ammonium recycling can be activated. This is sensible since conditions causing physiological low CO_2_ condition, e.g. drought, high temperature, *etc.* which causes stomatal closure (Ehleringer et al, 1991; Wilkinson and Davies, 2002; Sage et al., 2018), widely exist, mechanisms to enable capturing the released ammonium will confer competitive advantage for plants. This innate ammonium recycling mechanism can be used to solve the challenge of ammonium misbalance in C_2_ species when this is under demand after the re-allocation of GDC from MC to BSC (Mallmann et al., 2014). This finding is in line with many previous studies which demonstrate that C_4_ evolution recruited many pre-existing parts and regulatory mechanisms (Aubry et al., 2011; Reyna-Llorens and Hibberd, 2017; Dickinson et al., 2020), which together underlie the parallel independent evolution of C_4_ photosynthesis.

### Summary and perspective

Low CO_2_ is well recognized to be closely related to the evolution of C_4_ photosynthesis. Historically, this is mainly discussed from the perspective of low CO_2_ acting as a selection pressure. This study and also some earlier study show that leaves under low CO_2_ can also induce changes in the expression of many key C_4_ genes. Furthermore, here we show that low CO_2_ induces large scale rewiring of the primary metabolism to increase the capacity of leaves to assimilate ammonium, with ammonium accumulation being a possible candidate signal causing the observed changes. These innate mechanisms which induce the up-regulation of genes involved in ammonium refixation can be used to rebalance the ammonia residue after the GDC is reallocated from MC to BSC. Therefore, we propose that the mechanism responsible for the induction of genes involved in ammonium refixation represents a major regulatory mechanism, which facilitated the evolution of C_4_ photosynthesis.

Here we list some potential caveats of this study and should be the focus of future studies. First, we point out that during the evolution of C_4_ photosynthesis, there is a shift in the spatial expression patterns of critical genes involved in C_4_ photosynthesis (Reeves et al., 2017; Taniguchi et al., 2021). Whether low CO_2_ might have influenced the spatial expression pattern remains to be studied. Second, in this study, when the CO_2_ concentration decreased, the CO_2_:O_2_ ratio also decreased. Here we do not differentiate whether the observed responses is caused by the decreased CO_2_ level, or decreased CO_2_:O_2_ ratio. Thirdly, in this study, we show that the ammonium might be a potential signal inducing the observed changes in gene expression. However, under both low CO_2_ and high ammonium concentration, there is an increased carbon assimilation. The possibility of carbon deficiency as a signal for the observed changes also needs to be studied later. Fourthly, the signaling pathway underlies the observe changes in the gene expression needs to be elucidated as well. Elucidation of the detailed molecular mechanism will not only help understand how convergent evolution occurred for C_4_ photosynthesis, but also help guide future efforts of evolution guided engineering C_4_ prototype.

## Data availability statement

The RNA-seq datasets have been submitted to the NCBI Sequence Read Archive (SRA) under accession number PRJNA842829 (https://www.ncbi.nlm.nih.gov/sra/?term=PRJNA842829).

## Author contributions

X-GZ: Funding acquisition, Resources, Supervision, Writing – review & editing. FM: Conceptualization, Investigation, Writing – original draft, Writing – review & editing. YW: Writing – review & editing. NH: Writing – review & editing. M-JL: Writing – review & editing.
