# Supplementary material for: Rewiring of primary metabolism for ammonium recycling under short-term low CO2 treatment – its implication for C4 evolution
Source: Front Plant Sci. 2024 Aug 1;15:1322261. doi: 10.3389/fpls.2024.1322261 (PMC11324553; doi:10.3389/fpls.2024.1322261)
Supplement: Supplementary file 1 [file DataSheet_1.pdf]

Supplementary Information for

Regulatory preconditioning for the evolution of C_4 photosynthesis revealed by low CO_2 treatment of Arabidopsis thaliana

Fenfen Miao[1,2], Noor UI Haq[3], Ming-Ju Amy Lyu[2] and Xin-Guang Zhu[2,*]

[*]To whom correspondence may be addressed. Xin-Guang Zhu；
Email: zhuxg@sippe.ac.cn

This PDF file includes:

Table S1
Figures S1 to S5
Legends for Datasets S1 to S3

Other supplementary materials for this manuscript include the following:

Datasets S1 to S3

**Table S1 Gene-specific primers used for RT-qPCR**		
Gene	Forwards Primers（5‘-3‘）	Reverse Primers（5‘-3‘）
CA2	GAACGTATCGCTTGGAAACTTGCTT	TGGTCTTGAAATCGAGCTCCC
CA4	TGCTGCCCCAACTCAAAGTGAC	GCAAGTTTCCAAGCGATACGTT
CA5	TTCATTAGAAAGGCTGCTTGGGT	CGGTCTTTAACAGCGATTCCAC
PEPC1	CACATTGAAGAGAATCCGTGATCCG	CAGCAGCAATACCCTTCATCGTC
PEPC2	CGGCCACATCTCTCTAAGGAC	CATACCAGCAGCGATACCCT
AspAT2	CGCCAACAGTTATTTGAAGCTA	GGCACTGTCTTCGAACTTAGACC
AspAT5	CAAGAGCTGTATGATAGCCTCGTT	CATCAGCAAGATACTCGCATTTGG
AlaAT1	CGCCTTCTAAAAGCTACTGGAATAGTCG	CGGAACTCGTCCATGAAGCTC
mMDH	TCAGCTACATTGTCCATGGCCTA	CAAGCCTTCCTTCTCAAAGTCTG
pNAD-MDH	GCTACTTTGTCAATGGCTTATGCT	TCAATCACAGCTTCAAGACCGTTC
DIC	TTGCGAGTAATCCTGTTGATGTGA	TCGAAACCGTCGGGATAAAACCTT
DIT1	CCCACATTGGTGCTATGTTCACT	CATAGCCCCACCATTTAGCCAG
Fd-GOGAT	AGATCCAAAGAGTAACTGCGCCTG	TAAGCCGATTGAAATGTGACTTCC
GS2	GCACGAGACAGCTAGTATTGACCA	CTTTTCCTTTCGCCTCGGTGT
PEPC-K	ATATTCTTACGGAGAGAAGGTCGAT	AATCTTTAGCCATAGATGAAACCCC
PPDK-RP	TCGGAAAGAACTAGATTTCGCGTCA	GTCATGGTACAGCCGCAGAATCACA
ACT8	AGGGTTTCTCACTTCCACATGC	TCTCACAATTTCCCGTTCTGC

Table S1 Gene-specific primers used for RT-qPCR.

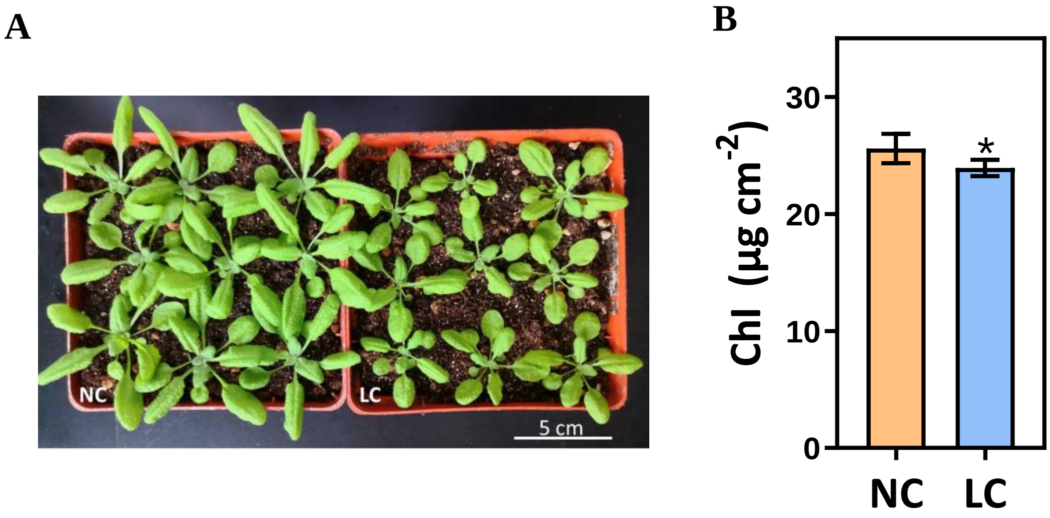

Fig. S1. Growth was arrested in *Arabidopsis* under low CO_2 conditions. (A) Phenotype, scale bars, 5 cm. (B) Chlorophyll content. Plants were grown for 21 days in normal CO_2 (400 ppm, NC) condition, then half of them were transferred to low CO_2 (100 ppm, LC) conditions for phenotyping and measuring. Data are shown as mean ± s.d (replications *n*=5), * p<0.05, two-sided Student's *t*-test.

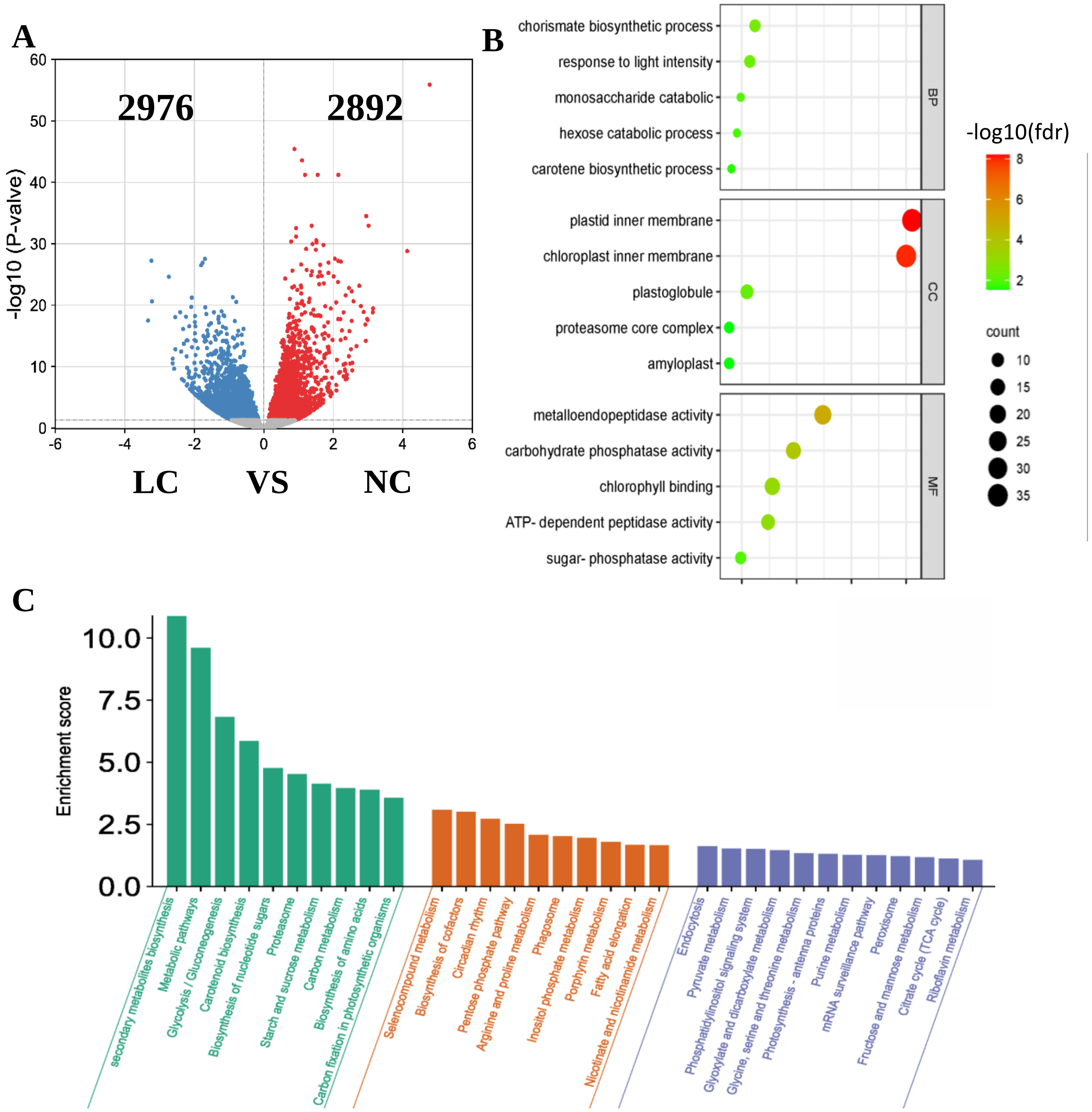

Fig. S2. Analysis of differentially expressed genes associated with low CO_2 treatment. (A) Volcano plot for the comparison between the low CO_2 treatment (LC，100 ppm) and normal CO_2 (NC， 400 ppm) in *Arabidopsis*. The cutoff values *adjusted P-value* <0.05 were utilized to identify differentially expressed genes. Non-changed genes were shown in grey color. The red color is indicative of upregulated genes and blue is indicative of downregulated genes. (B) GO enrichment of differentially upregulated expressed genes under low CO_2 treatment. (C) KEGG enrichment of differentially up-regulated expressed genes under low CO_2 treatment.

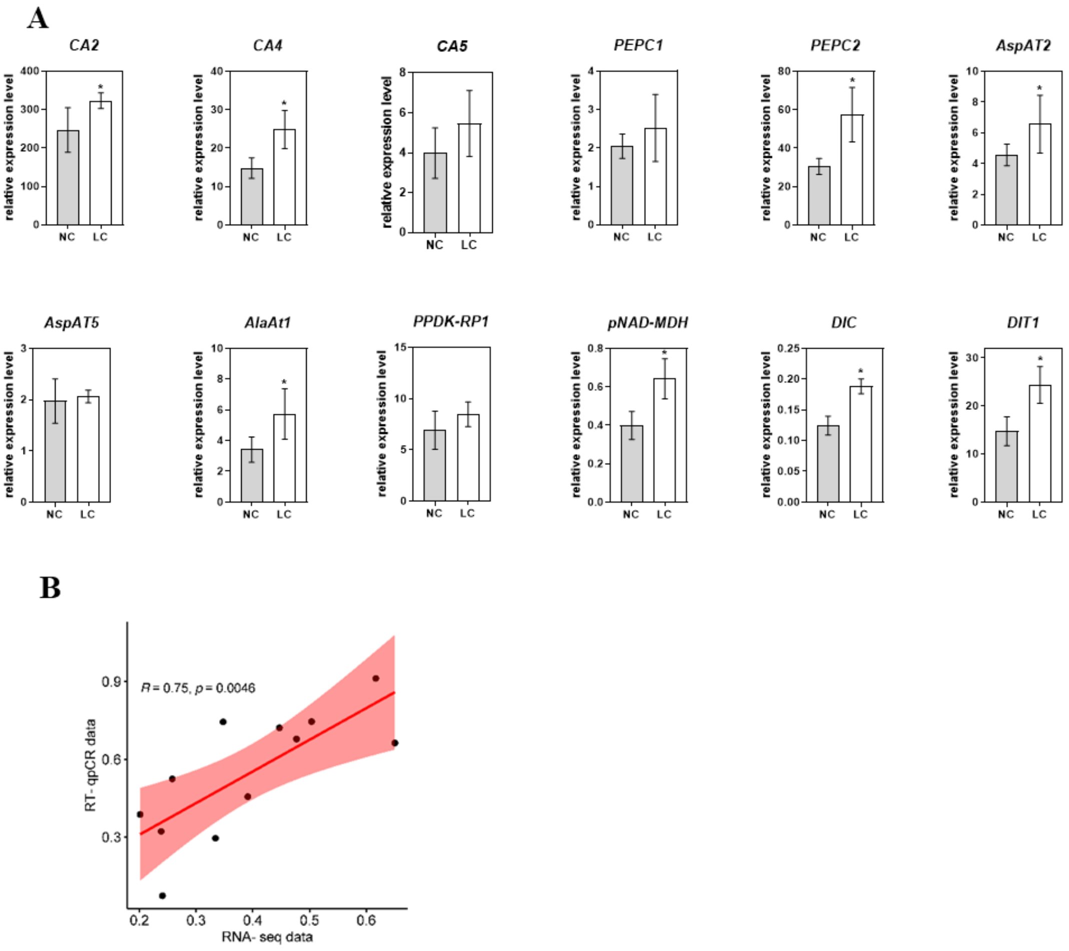

Fig. S3. Related genes were verified by RT-qPCR. The abbreviations are as Fig. 1. Data are shown as mean ± s.d (replications n=4), * p<0.05, two-sided Student's t-test.

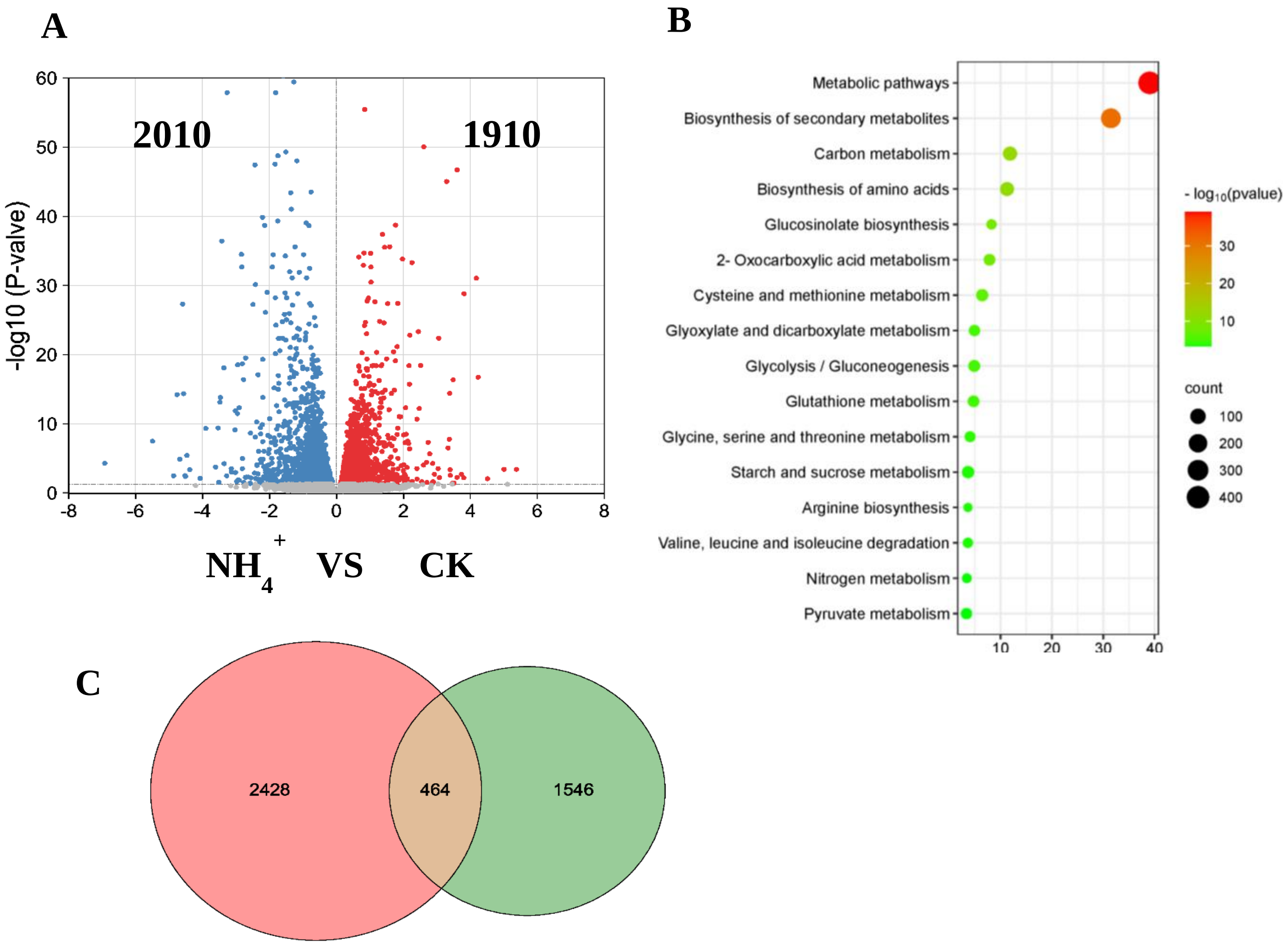

Fig. S4. Identification of differentially expressed genes associated with NH_4^+ treatment. (A) Volcano plot for the comparison between 30 mM NH_4^+ (NH_4^+) and control check (CK) in *Arabidopsis.* The cutoff values *adjusted P-value* <0.05 were utilized to identify differentially expressed genes. Non-changed genes were shown in grey color. The red color is indicative of up-regulated genes and blue is indicative of down-regulated genes. (B) KEGG enrichment of differentially up-regulated expressed genes under NH_4^+ treatment. (C) Overlap of jointly downregulated genes after low CO_2 treatment and NH_4^+ treatment. Detailed gene information can be found in Dataset S3.

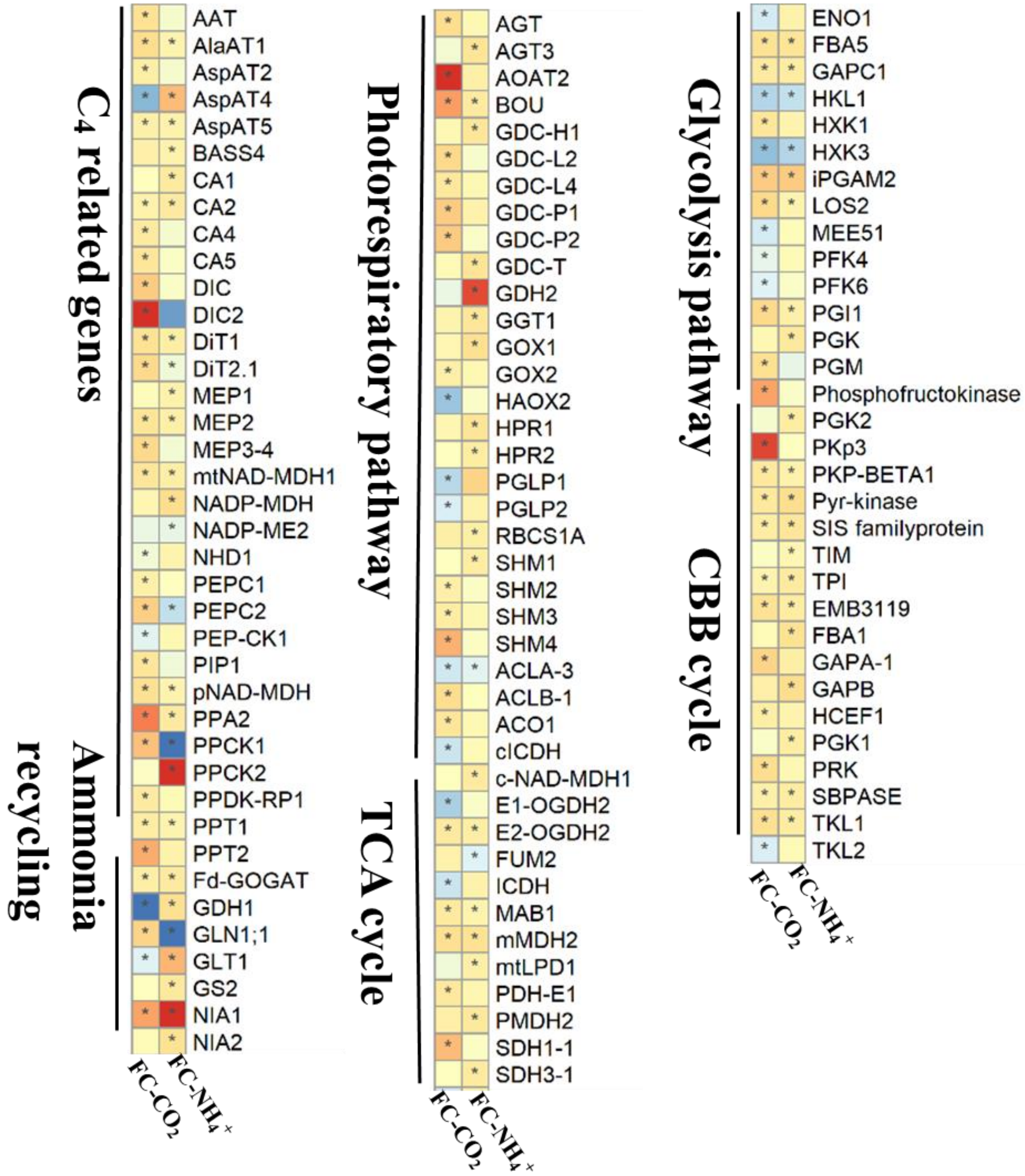

Fig. S5. The comparison of gene expression between low CO_2 treatment (FC-CO_2) and NH_4^+ (FC- NH_4^+) treatment in different metabolic pathways. The symbols of genes in these metabolic pathways are from the Arabidopsis Information Resource (TAIR) Database (http://www.arabidopsis.org/), and also can get from Dataset S1.

Supplementary data

Datasets S1. The differentially expressed genes (DEGs) of transcripts and primary metabolism after low CO_2 and NH_4^+ treatment, and the changes in metabolic profiles under low CO_2 treatment.

Datasets S2. GO and KEGG enrichment analysis after low CO_2 and NH_4^+ treatment.

Datasets S3. Overlap of DEGs after low CO_2 and NH_4^+ treatment and their KEGG and GO enrichment data.
